# Emerging Frameworks in Health and Education: Conceptual Evolution in Pursuit of School-Based Health

**DOI:** 10.5334/cie.62

**Published:** 2023-02-16

**Authors:** Daniel Skinner, Hannah Palme’, Sara Bode, Mary Kay Irwin

**Affiliations:** 1 Ohio University, Heritage College of Osteopathic Medicine, US; 2 Nationwide Children’s Hospital, US

**Keywords:** school-based health, Alma-Ata, Whole School, Whole Community, Whole Child

## Abstract

This paper presents a historical account of conceptual development at the intersection of American education and health. Beginning with early advancements from the Association for Supervision and Curriculum Development and the World Health Organization, the authors show the movement from early considerations of the codependency of health and education. The authors suggest that more than fifty years of theoretical innovations at the nexus of health and education culminated in the 2014 introduction of “Whole School, Whole Community, Whole Child” (WSCC). At the same time, the authors show that the trajectory of this movement was far from linear. In addition to explaining why WSCC is in many ways a critical revision of the social determinants model that serves today as a promising foundation of American school-based health, the authors examine opportunities and challenges that the pivot towards WSCC presents. Three particular areas are explored: assessment, funding, and collaboration.

Conceptual evolution sits at the center of scientific progress, and this is true of population health as well. Over the past decades the science of health, wellness, and disease prevention have undergone continual conceptual change with great benefits to health outcomes. And yet, we also know that conceptual evolution is often a long and hard road. As the physicist and philosopher Thomas Kuhn famously argued in the 1960s, scientific revolutions are not purely rational, linear movements driven by the latest and best available evidence (Kuhn, 1962). For Kuhn, “paradigm shifts” such as the one we describe are often slowed by tensions within different institutional norms, objectives, as well as interests. They are also undermined by territorialism and competitiveness. The very name “school-based health” points to the complexity of the question. Not only do school-based health programs seek to leverage schools as key sites for promoting health, but these programs must find creative ways of bridging both health and educational institutional and professional cultures.

This paper presents a historical account of the evolution of conceptual frameworks at the intersection of American education and health. Our particular focus is the rise of school-based health. This almost 50-year history has developed in ways that Kuhn would have predicted. At the same time, this history is also driven by unique forces that have helped it to evolve, culminating, we argue, with the introduction of the “Whole School, Whole Community, Whole Child” (WSCC) framework in 2014. This framework, the deeper conceptual evolution of which scholars have yet to document, provides a lens for understanding the benefits of school-health collaborations, as well as, providing both educational and health experts with a roadmap for doing so. In addition to explaining why WSCC is in many ways a critical revision of Social Determinants of Health models that are commonly discussed in health care, we examine some of the opportunities and challenges that the pivot towards WSCC presents for public health advocates, scholars, and members of both healthcare and educational institutions.

## A Brief History of Frameworks in Education and Health

Though often encountered together today, conceptual frameworks shaping health and education have had both a complicated and often divergent history. To understand the development of these entwined frameworks, it is important to consider key steps in this history, tracing points of origin and interconnection.

### Early Influences

The first key development came in 1943 when the Association for Supervision and Curriculum Development (ASCD) was created through a merger between the National Education Association and the Society for Curriculum Study and Department of Supervisors and Directors of Instruction. The formation of the ASCD was the result of a belief that “rigid hierarchies” were impeding efforts to envision a more holistic approach to education, especially one that considered the relationship between child health and development (ASCD, 2018). Following World War II, global public health organizations began taking an interest in education. In 1950 the World Health Organization (WHO) created the Expert Committee on School Health Services composed of experts including academics, medical officers, and school nurses (World Health Organization, 1951). The goal of this meeting was to encourage health and education systems to work as a collective to promote health, as well as, to make policy recommendations to improve the health of children and communities (World Health Organization, 1997).

Throughout the 1950s and 1960s, both the education and health sectors underwent significant evolutions in their thinking, with landmark documents charting the evolution of the frameworks within which they were increasingly working. Building on this prior call for collaboration, in 1966 WHO published one of the first international plans for incorporating health into schools, based largely on epidemiological data. Among other things, the plan underscored the importance of basic health indicators for schools’ aims. As ideas behind education and health were evolving, a “humanist” movement was also making an impact, especially among the leaders of the ASCD. In 1962 the ASCD officially joined this movement and began advancing curricular ideas that reflected values of personal fulfillment, including health, education, nutrition, and exercise (Van Til, 1986). These two decades included a myriad of influential conferences at which ideas were hashed out, leading to an infusion of new scholarly frameworks.

The early 1970s saw groundwork laid for what is now known as the “social determinants of health” (SDOH). In 1974 the Canadian Department of National Health and Welfare released *A New Perspective on the Health of Canadians: A Working Document*, better known as the “Lalonde Report,” which identified lifestyle, environment, healthcare organization, and human biology as integral to population health. The report marked the early stages of development of the concepts of preventive health and health promotion and led to a wave of new research related to the intersection of child development and education (Glouberman and Millar, 2003).

The Lalonde Report’s influence was felt acutely at the International Conference on Primary Health Care in 1978 with the Declaration of Alma-Ata, which identified “primary health care” as a key to health promotion (World Health Organization, 1978) and was hailed as “the first international attempt at this level to identify the myriad of factors influencing health” (St Leger, 1999). In 1986, in recognition of the importance of social determinants and health education, WHO and the Canadian government hosted the 1st International Health Conference in Ottawa. Building on Alma-Ata, the Ottawa conference introduced the Global Strategy of Health for All by the Year 2000, another milestone call to address policy at all levels including food, education, and shelter to promote health (World Health Organization, 1986).

### American Frameworks

In 1979, the year after Alma-Ata, a Surgeon General’s Report on Health Promotion and Disease Prevention emphasized the role of schools in promoting health and health education in the nation’s youth and underscored the importance of a basic understanding of health and disease for child development (Office of the Surgeon General, 1979). Although this report was an important step in the history and evolution of U.S. health education, in 1984 ASCD pointed to a failure to provide adequate financial support to implement the recommendations they themselves had proposed (Van Til, 1986). As before, critical conceptual developments lacked formal collaboration and support between educational and health organizations, and the U.S. government did not arise as a convener of private and public sector entities.

The specifically American part of this evolution toward unified frameworks in education and health began in 1987 when the Centers for Disease Control and Prevention (CDC) created the Coordinated School Health Program, which encouraged health services in U.S. schools, with a focus on community engagement and family involvement (Rasberry, et al, 2015). This period, which stretched into the 1990s, saw an increased focus on the needs of students through integrated and coordinated school and community partnerships, as well as, the documentation of outcomes. In 1995, for example, new national health education standards provided benchmarks for Health Education in Pre-K through 12th grade (Centers for Disease Control, 2019). This accountability structure validated the need for school-age children to learn health promotion and created a framework for approaching both curriculum and instruction. However, it was not until 2007 that American schools began, at least on a large scale, to create and adopt such frameworks. Integrated frameworks, not to mention school-based health itself, remained primarily local. Though no in-depth studies have yet sought to understand the nature of the barriers that delayed the arrival of these new integrated frameworks, the absence of meaningful partnerships, especially from the health side, and a “go at it alone” mentality on the part of educational institutions seem to have played important roles.

### Enter “Whole Child”

In 1997, Dr. Hiroshi Nakajima, Director-General of the WHO, encapsulated the conceptual progress that had been made regarding the importance of leveraging schools in the pursuit of global health. As Nakajima noted:

Health is inextricably linked to educational achievement, quality of life, and economic productivity. By acquiring health-related knowledge, values, skills, and practices, children can be empowered to pursue a healthy life and to work as agents of change for the health of their communities (World Health Organization, 1997, p.1).

Nakajima’s words are quoted often, and the WHO report for which they serve as an epigraph provides a clear rationale for health education standards in schools. But it also goes beyond a rationale, suggesting that if students are not healthy, they will not be able to achieve their educational potential–and vice versa. Education and health, in other words, are inextricably intertwined and codependent.

By the mid-to-late-2000s, this idea had become increasingly accepted as a cornerstone of both educational and health theory. Critical to the intellectual debates taking place in the U.S. at the time was the educational climate created by the “No Child Left Behind” Act (NCLB), which was widely criticized for focusing on academic achievement without attending to critical intervening and cross-cutting factors. Two scholars of the bill claimed that:

The NCLB is largely predicated on the unfounded assumption that the educational gap can be closed without addressing gaps in child development and physical and mental health that affect learning. It fails to sufficiently invest in crowded urban and rural schools serving poor and minority children. It represents a largely unfunded, and arguably unattainable, mandate to states, school districts, and local public schools (Fiscella and Kitzman, 2009).

At this time, in 2006, the ASCD created the Commission of the Whole Child, consisting of experts and leaders in the field, to rethink the definition of a successful student. A key outcome of the Commission’s work was the Whole Child Model (WCM), defined as “an effort to change the conversation about education from a focus on narrowly defined academic achievement to one that promotes the long-term development and success of children” (Association for Supervision and Curriculum Development, 2015). Whole Child was in many ways a path breaking approach to the education of children that remained focused on keeping students healthy, safe, engaged, supported, and challenged. By 2015, Congress replaced NCLB with new legislation known as the “Every Student Succeeds Act,” which called for a “well-rounded education,” that was interpreted as a signal of an openness to a broader approach to education, and one that could include health.

### From Whole Child To WSCC

Whole Child’s arrival set new terms of debate. Despite the fact that both education and health scholars had by now long acknowledged the importance of considering both the health and education of American youth, a clear and common goal proved elusive until 2014 when a collaboration between the CDC and ASCD led to the creation of WSCC.

WSCC, which was presented at the annual ASCD Whole Child Symposium in 2014 (Association for Supervision and Curriculum Development, 2014), was essentially a distillation of key points from the WCM and CDC’s Coordinated School Health (Centers for Disease Control and Association for Supervision and Curriculum Development, 2014). WSCC’s goals were laid out in a report entitled *The Whole School, Whole Community, and Whole Child: A Collaborative Approach to Learning and Health* published in 2014 by the ASCD in collaboration with the CDC. WSCC outlined a host of non-academic barriers that impede learning and present challenges for educators tasked with helping their students experience optimal education outcomes. Like SDOH, WSCC identified key barriers related to stable housing and transportation, financial security, connectedness to community, and reliable access to healthy food. Beyond the stated aims of SDOH, WSCC added an additional and key point that these determinants needed to be addressed for children to learn. WSCC echoed the SDOH insistence that education was a key driver of health outcomes, including disparities, but took an additional step of identifying health status as a primary influencer of educational attainment (Lewallen, et al, 2015). WSCC cast traditional educational metrics such as daily attendance, the need for disciplinary actions, academic progress measured by standardized testing, kindergarten readiness, and high school graduation rates as functions of non-academic barriers that required broad and collaborative attention (Centers for Disease Control and Association for Supervision and Curriculum Development, 2014). As such, WSCC raised the question of assessment while pointing to a broader set of areas in which relevant assessment data could–and must–be identified.

WSCC’s overarching goal was “greater alignment,” “integration.” and “collaboration” between educational and health entities (Chiang, Meagher, and Slade, 2015). For example, it is widely accepted that students who are frequently absent from school encounter more barriers to achievement and health concerns than students who routinely attend school (Allison, et al, 2019). Accordingly, both WSCC and SDOH not only identify health-related issues as primary drivers of absenteeism but establish timely access to healthcare–from routine vaccinations to comprehensive annual exams–as priorities. In addition, in clear alignment with SDOH, WSCC specifically recognized that achieving optimal health outcomes requires that healthcare professionals look beyond a given health condition to address external barriers that influence the health and well-being of their patients. WSCC acknowledged that achieving optimal education outcomes also requires that educators identify external factors that are negatively impacting student attainment. Health became a natural and core focus.

## The (Slow) Turn Toward Population Health

While we have narrated key historical moments in the preceding sections, it is important to supplement that history with a description of macro-level changes in health care itself that serve as a critical background for this work. After all, as Kuhn taught us to expect, the force of ideas alone is rarely sufficient in bringing about structural change. The good news is that the decades of scholarly advancements in this area point to areas in which smart investments can be made, beyond their often insular and contained campuses to include mobile units, a range of new outpatient facilities, but also to make new in-roads in institutions as diverse as religious institutions and schools.

It’s important to note that the past few decades have also seen the beginnings of movement away from “fee-for-service” healthcare payment models in which healthcare providers are paid for individual tests and examinations instead of outcomes (Schroeder and William, 2013). Though fee-for-service remains the dominant payment model, with alternatives slow to come (Zuvekas and Cohen, 2016), scholars have tended to miss the important relationship between the financing of health care and the attractiveness of school-based health centers. The critical shift away from fee-for-service healthcare financing has led health policy and health services researchers to increasingly champion population health-sensitive models (especially accountable care organizations) that compel healthcare institutions to think creatively about how to become institutions of prevention instead of mere illness.

The specific mechanism for this development is the incentivization of healthcare institutions to take responsibility for the health of specific populations. These incentives included financial rewards in instances where more efficient healthcare delivery yields either the same or better outcomes. A key effect of this movement was making clear that the costs of inefficiencies, such as preventable emergency room visits, would be borne by the institutions themselves. These financial arrangements created powerful incentives for hospitals and healthcare systems to be creative, moving beyond hospital walls to take a more proactive approach toward health (Perrin, et al, 2017). In the case of safety net facilities, from children’s hospitals to Federally Qualified Health Centers, which are often largely dependent on managing Medicaid payments efficiently, this logically led to a realization that schools were important spaces for the early identification of risk signs, as well as a high impact “upstream” site for intervention with significant “downstream” effects (Williams, et al, 2008).

Within a renewed focus on outcomes, we can also point to important examples of local, state, and federal resources brought to bear to support the kind of school-based health programs that embody the WSCC vision. All are part of an increasing investment in strategies that think holistically about children’s health and key drivers of outcomes, with schools identified as high yield sites of engagement. For example, the state of Ohio’s Student Success and Wellness Funds serve as a model of education funding to support the integration of health and wellness activities in schools. Similarly, Ohio’s “Medicaid School Program,” a joint effort of the state’s Department of Medicaid and Department of Education, allows schools to bill Medicaid for services such as school-based occupational therapy, physical therapy, and some school nursing services (Ohio Department of Education, no date). Another resource stream comes from the American Rescue Plan Act of 2021, better known as the COVID-19 Stimulus Package, which provided extensive funds to American schools with a requirement that funds be spent to address issues created by the pandemic. It is still unclear whether and to what extent these funds will be converted into high yield programming, such as tutoring resources to aid students whose education was delayed by the pandemic or hiring more school nurses or contact tracers. While the possibilities are many, the key point is that such investments in health are now widely believed to have a direct impact on educational attainment, and vice versa. In other words, they are considered to be consistent with the missions of both educational and healthcare institutions.

## Challenges

The emerging culture within both schools and healthcare institutions will certainly help create increasingly hospitable environments within which the values embodied by WSCC can continue to evolve and grow. At the same time, a series of considerations continue to complicate and challenge.

### Assessment

Parallel to the history we narrated above, tools were developed to guide needs assessments of WSCC implementation plans. Among these tools were the CDC’s School Health Index, a series of modules developed from CDC’s Coordinated School Health Program model that describes healthy lifestyle behaviors and seeks to reduce health-related risk behaviors (Pearlman et al, 2005), and the ASCD School Improvement Tool, a free online needs assessment survey that helps schools to integrate WSCC (ASCD, no date). During the period in which these new ideas were being introduced into mainstream educational thinking, objectives associated with SDOH became standard in nearly every community health assessment and were included in health improvement initiatives such as the Healthy People report released each decade by the U.S. Department of Health and Human Services (Office of Disease Prevention and Health Promotion, 2020).

Despite these steps, assessment remains a challenge. Though shared accountability opportunities are emerging slowly, healthcare providers are still largely unable to determine if health-related services delivered at school improve attendance (a short-term goal) or attainment (a long-term goal) without improved access to educational data. Educators are equally challenged to assess their effectiveness in contributing to improved health and wellness of their students without access to health-related data. The problem is that existing assessment tools tend to be tailored to either assist an educational or healthcare team in an evaluation slanted towards their respective discipline or environment, rather than taking a truly multidisciplinary team perspective. Both WSCC and SDOH emphasize the interdependence of health and education and the criticality of collaborative strategies across disciplines. Unfortunately, these programs have suffered from inadequate evaluation, in part because existing assessment tools have tended to be poorly designed for understanding the intersection of health and education.

In rare instances in which professionals attempt to establish multidisciplinary evaluation plans to assess the effectiveness of WSCC implementation, these collaborations face additional challenges. Linking health and education outcomes requires sophisticated legal agreements permitting stakeholders to share data that is protected by different privacy laws (including the Health Insurance Portability and Accountability Act of 1996 within health care and the 1974 Family Educational Rights and Privacy Act for educators). If teams are successful in executing data-sharing agreements, the actual process of linking data can be complicated by logistical barriers such as obtaining consent and matching medical record numbers with student ID numbers. Another barrier concerns the inability to isolate factors contributing to outcomes. By design, WSCC is intended to address multiple factors that impede learning by providing “wraparound” supportive interventions. With the implementation of multiple interventions and interconnected supports at the same time, researchers struggle to identify which interventions are effectively creating positive impact.

### Funding

Though we have described a few helpful funding streams above, especially on the state level, WSCC’s history has reminded us of the chasm that often exists between the advancement of new ideas and securing resources to put them into practice. Funding to support WSCC activities varies greatly between states. In the most recent report (Fiscal Year 2017) of the School-Based Health Alliance, which conducts a triennial survey to assess policies and available resources supporting the advancement and sustainability of school-based health centers, only 16 states plus the District of Columbia reported dedicated investments (School-Based Health Alliance, no date). In the absence of state funding, healthcare providers struggle to cover start-up costs to renovate space, purchase equipment, and upgrade infrastructure. Many American schools are old or aging (Vincent, 2006), which impacts not only the availability of new spaces for health promotion, but presents costly challenges in the pursuit of effective and compliant technological solutions to operate medical equipment and to access electronic health records. Although most school-based healthcare providers bill for their services, it can be years before a school-based health center reaches maturation and is caring for enough patients to cover expenses.

In the case of WSCC, however, there is reason to be optimistic that such resources are forthcoming. As we’ve noted, schools are now increasingly regarded as obvious sites for carrying out population health initiatives, which suggests that fissures and differences in institutional cultures will eventually be worked through. One recent acknowledgement of the value of this work and the understanding that it requires financial resources is the inclusion of provisions in the American Rescue Plan Act to support school-based mental health systems, overall health and wellness initiatives, and wrap-around services for homeless children and youth (US Department of Education, 2021). The allocation of government resources to support interventions aligned with WSCC will not only result in timely, new school-based services, but will also likely create an awareness of need that hopefully generates more funding streams in the public and private sectors.

### Collaboration

A related challenge concerns the delineation of roles between healthcare providers and school personnel. Champions of WSCC cannot afford to downplay the challenges of actualizing the culture of collaboration necessary to integrate schools and health care. For example, to realize WSCC’s potential, it is important to understand the different roles of school counselors in contrast to behavioral health therapists, school nurses, and advanced practice nurses, on the one hand, and community health education experts and health teachers, on the other. The good news is that each of these long-established positions has a unique contribution to make within the spaces in which school-based health is carried out. But it is logical that integrated frameworks might stoke fears of job insecurity and arouse territorialism, just as they often have in interprofessional developments in health care more generally (Axelsson and Axelsson, 2009). Historically, for example, many school nurses are part of teachers’ unions, which makes union negotiations and related issues of job security part of the broader conversation about roles and procedures. Attending to these important historical and institutional differences can prevent the rise of unintentionally competing accountability structures.

## Conclusion

The story of conceptual development leading to WSCC was one of working through shared missions and interests that have long existed but had been kept at a distance because of the distinct histories that American schools and healthcare institutions have had. It makes sense that this history would be disjointed instead of a smooth, linear trajectory. While we have suggested that larger trends in health care, where financial incentives play a large and important role, can accelerate innovation, it is also true that the work ahead to actualize WSCC’s aims will be difficult, slow, and uneven. Far from suggesting that this conceptual development is complete, or has reached an apex with WSCC, the challenges we have identified are significant. As Kuhn predicted regarding revolutions in scientific thinking generally, one should anticipate resistance.

It is clear, however, that the aligning interests of both schools and healthcare institutions are creating an increasingly hospitable environment for not only the continuation of the conceptual evolution we have described, but its entrenchment. Schools are well-positioned to play a critical role, particularly as they are overwhelmed by the growing complexity of issues impeding their students’ ability to learn and are desperate for resources and support to address the nonacademic barriers to learning. Given the centrality of schools in communities, coupled with the fact that school is the one place that children predictably frequent, schools are uniquely positioned to serve as the primary convenor for this type of collaboration. Healthcare providers, for their part, are equally challenged to improve the health outcomes of their patients and are motivated to identify nontraditional mechanisms of care to address the barriers impeding overall health and well-being. Schools are now widely understood to be critical sites for health prevention and intervention. With educators and healthcare providers at the ready, advocates should focus on the identification of resources needed to facilitate this type of collaboration.
